# Feasibility and effectiveness of hysteroscopic myomectomy for type 3 myomas

**DOI:** 10.3389/fsurg.2025.1742020

**Published:** 2026-01-05

**Authors:** Hui Yan, Junwei Li, Tingting Wang, Zhong Liu

**Affiliations:** Department of Obstetrics and Gynecology, The First Affiliated Hospital of Ningbo University, Ningbo, Zhejiang, China

**Keywords:** fibroids, hysteroscopy, menorrhagia, myomectomy, type 3 myoma

## Abstract

**Objective:**

This study aimed to evaluate the feasibility and effectiveness of hysteroscopic myomectomy for treating International Federation of Gynecology and Obstetrics type 3 myomas.

**Methods:**

This retrospective study evaluated 26 patients with type 3 myoma who underwent hysteroscopic myomectomy in a teaching hospital between January 2022 and May 2025. Data on surgery time, transurethral resection of prostate syndrome, and complete removal rate were collected. The amount of menstruation which was assessed by pictorial blood loss assessment chart (PBAC). The PBAC, duration of menstrual period, and hemoglobin levels were recorded at 3, 6, and 12 months postoperatively.

**Results:**

The complete myoma removal rate was 96.15%. One patient developed transurethral resection of prostate syndrome, while no cases of postoperative intrauterine adhesion were detected on three-dimensional (3D) transvaginal ultrasound. A significant reduction in menstrual blood loss was observed after the procedure. Notably, three women conceived naturally within 1 year postoperatively.

**Conclusion:**

Hysteroscopic myomectomy is a viable treatment option for International Federation of Gynecology and Obstetrics type 3 myomas and can effectively relieve menorrhagia in highly selected patient population.

## Introduction

Uterine myomas are the most common neoplasms of the female reproductive system and can adversely affect fertility, with an incidence ranging from 217 to 3,745 cases per 100,000 women-years and a prevalence of 4.5%–68.6% (1). In earlier classifications, myomas were broadly categorized as submucosal, subserosal, and intramural myomas. In 2009, Pritts et al. reported that subserosal myomas had no impact on fertility outcomes, submucosal myomas negatively affected fertility, and intramural myomas appeared to reduce fertility, although treatment outcomes remained unclear (2). A high concentration of proinflammatory nuclear factor kappa B and low levels of homeobox and leukemia inhibitory factor mRNA expression were detected in patients with type 0 or 1 myomas. These levels returned to normal following hysteroscopic myomectomy (3). In 2011, the International Federation of Gynecology and Obstetrics (FIGO) introduced a new classification system that divided uterine myomas into nine types based on their location within the uterus (4). Initially, type 3 myomas were categorized as intramural myomas, as they were considered completely extracavitary. However, due to their contact with the endometrium (4), type 3 myomas were reclassified as submucosal in the 2018 FIGO update. Type 0–2 myomas were known to adversely impact fertility due to their distortion of uterine anatomy. The effect of intramural myomas, particularly non-cavity-distorting ones, on fertility had recently gained attention. These myomas have been associated with reduced rates of live birth, clinical pregnancy, implantation, and delivery in patients undergoing assisted reproductive technology (5). Furthermore, the removal of asymptomatic, cavity-distorting myomas had been shown to improve fertility outcomes and/or reduced miscarriage rates (6). In 2016, Capmas et al. (7) suggested that hysteroscopic resection of type 3 myomas could be a viable alternative to traditional surgery, provided it is performed by experienced surgeons. Type 3 myomas negatively affected implantation, clinical pregnancy, and live birth rates in patients undergoing *in vitro* fertilization–intracytoplasmic sperm injection, although they did not significantly increased the rate of clinical miscarriage. Additionally, type 3 myomas with a total myoma diameter or a single myoma diameter >2.0 cm had been associated with a more pronounced decline in implantation, clinical pregnancy, and live birth rates among patients undergoing *in vitro* fertilization–intracytoplasmic sperm injection (8). One study found that women with FIGO type 3 intramural myomas had significantly lower implantation rates (IPR) (15.7% vs. 24.6%, *p* = 0.015), clinical pregnancy rates (CPR) (23.7% vs. 38.1%, *p* = 0.014), and live birth rates (LBR) (16.5% vs. 30.4%, *p* = 0.011) compared to women without myomas (9). These reduced IPR, CPR, and LBR indicate that type 3 myomas could have a detrimental impact on IVF outcomes, with the severity of these effects increasing alongside the size and number of myomas (10). However, the benefits of surgical intervention for FIGO type 3 myomas on *in vitro* fertilization outcomes remain unclear (10). Limited evidence suggested that hysteroscopic myomectomy is a safe and feasible option, although studies to date have involved only small patient cohorts (11). Therefore, further research is needed to evaluate the safety and efficacy of hysteroscopic treatment for FIGO type 3 myomas (11). This study aim to assess both the feasibility and effectiveness of hysteroscopic myomectomy in this context.

## Methods

### Patients

This retrospective study evaluated the clinical effects of hysteroscopic myomectomy on patients with FIGO type 3 myomas who underwent hysteroscopic myomectomy at the First Affiliated Hospital of Ningbo University between January 2022 and May 2025. During this period, there were 50 cases of soley type 3 uterine myomas confirmed by 3D transvaginal ultrasound. 11 patients chosed for laparoscopic myomectomy and 5 refused surgical intervention due to normal menstrual. 34 patients were underwent hysteroscopic myomectomy. Two patients were postmenopausal and were excluded due to the inability to accurately perform endometrium-preserved hysteroscopic surgery. Among the remaining cases, 6 cases were identified as type 2 myomas intraoperatively and excluded from the study. 26 patients were considered eligible.

The following clinical data were recorded: age, symptoms, size of myoma, preoperative sonography findings, the menstrual volume, duration of the menstrual period, preoperative hemoglobin (Hb) level, number of surgeries performed for resection, surgery duration, and complications (intravasation syndrome and perforation). The menstrual volume was evaluated by the pictorial blood loss assessment chart (PBAC) score. In this method, light flow was defined as staining covering ≤1/3 of the sanitary pad area, moderate flow as staining covering 1/3–3/5 of the pad area, and heavy flow as extensive staining covering most of the pad area. Corresponding scores were 1, 5, and 20, respectively. The total PBAC score was derived by summing the sanitary napkin score and the clot score, with a threshold of ≥100 points indicating menorrhagia (corresponding to >80 mL blood loss). Routine 3D ultrasonography was conducted postoperatively to assess for residual myoma. Follow-up evaluations were performed at 3, 6, and 12 months.

This study was approved by the Ethics Committee of the First Affiliated Hospital of Ningbo University (Zhejiang, China).

### Hysteroscopic myomectomy of type 3 myoma

Hysteroscopic myomectomy was performed with patients in the lithotomy position. The cervix was gently dilated using Hegar dilators, gradually increased in size until it could accommodated a size 10 dilator. The slicing technique is commonly used to remove type 3 myomas. During the whole surgical procedure, we utilized 26-Fr hysteroscope with a 30° viewing angle (Olympus Co., Tokyo, Japan) with the plasma bipolar cutting loop. The saline (0.9%) was used as the distention medium. To make resection easier, the electrosurgical loop was set at 200 W in cutting mode and 120 W in coagulation mode. All surgeries were performed by a skilled gynecologist who had completed >500 hysteroscopic procedures per year.

**Figure 1:**
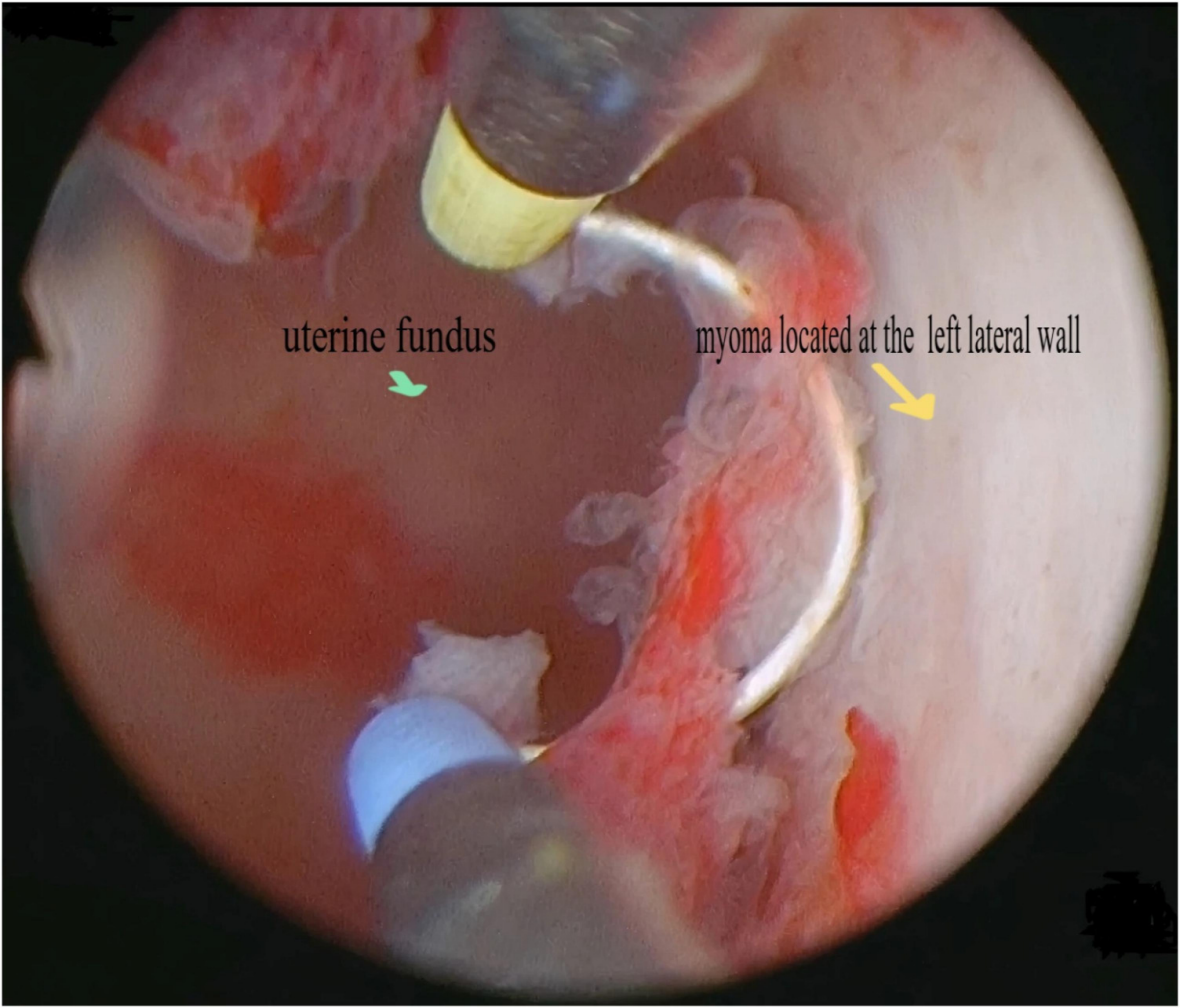
The uterine cavity without deformation with minimal pressure.

**Figure 2:**
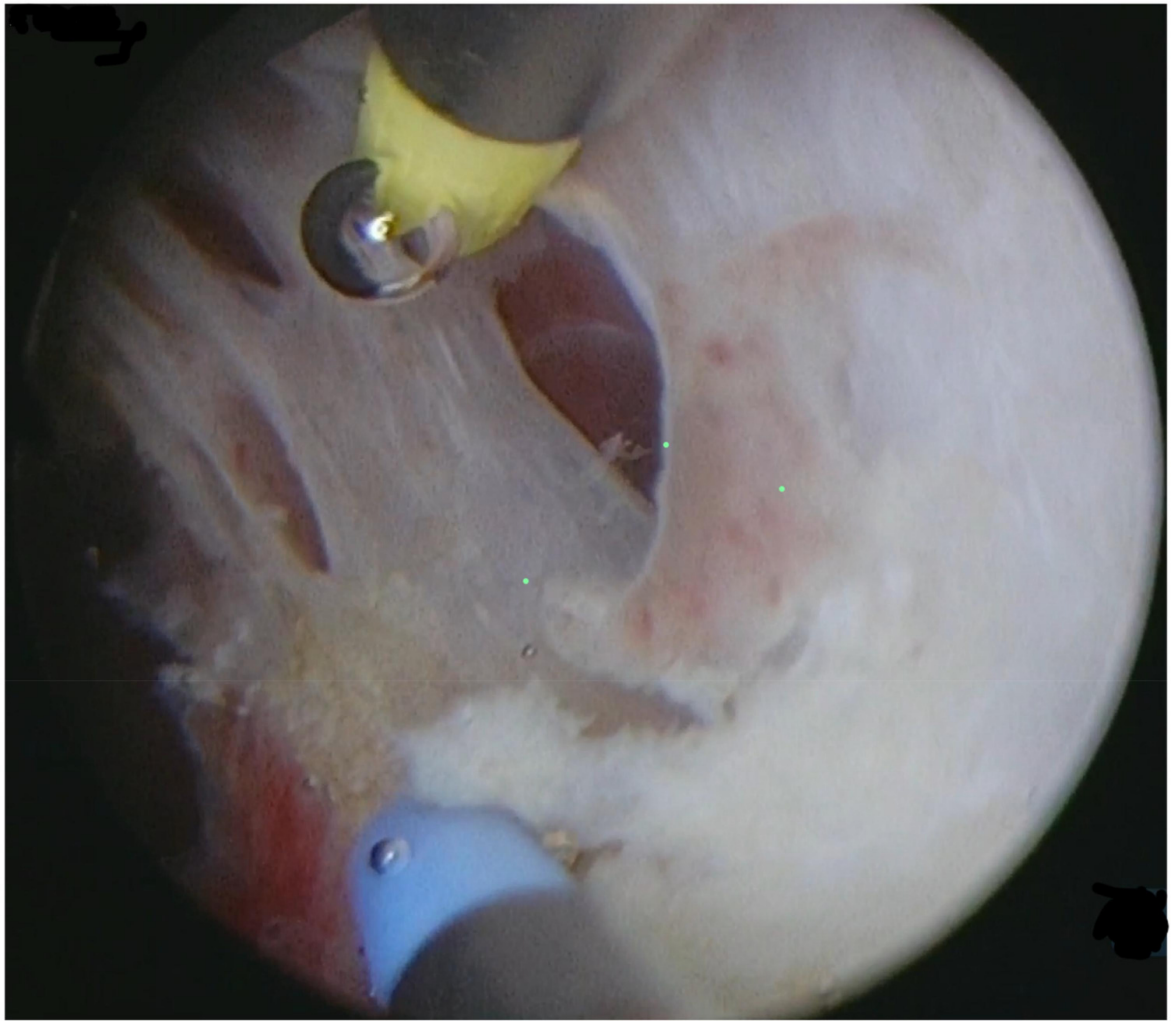
Opening the pseudocapsule of the type 3 myoma.

**Figure 3:**
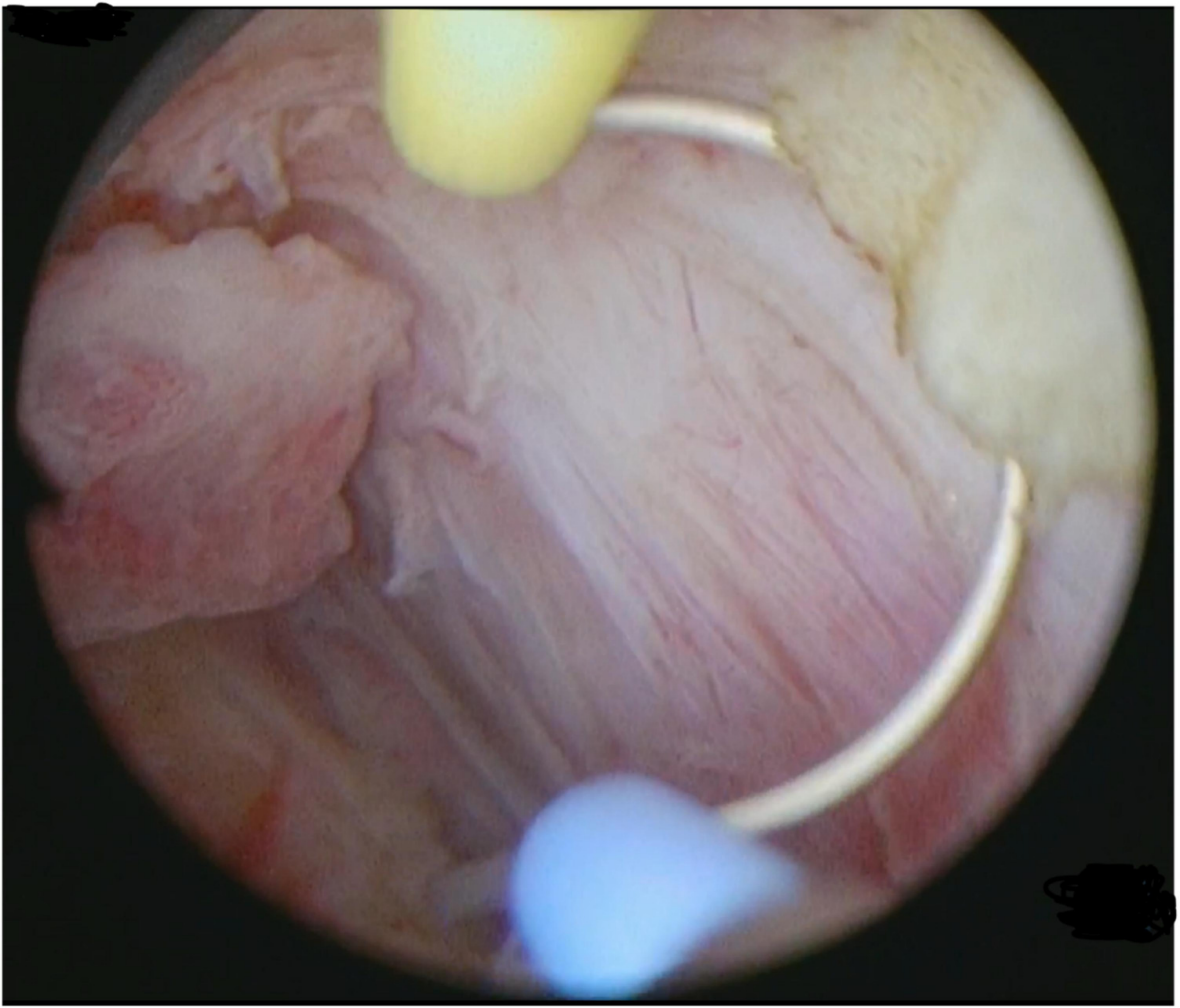
Type 3 myoma was converted into type 2.

**Figure 4:**
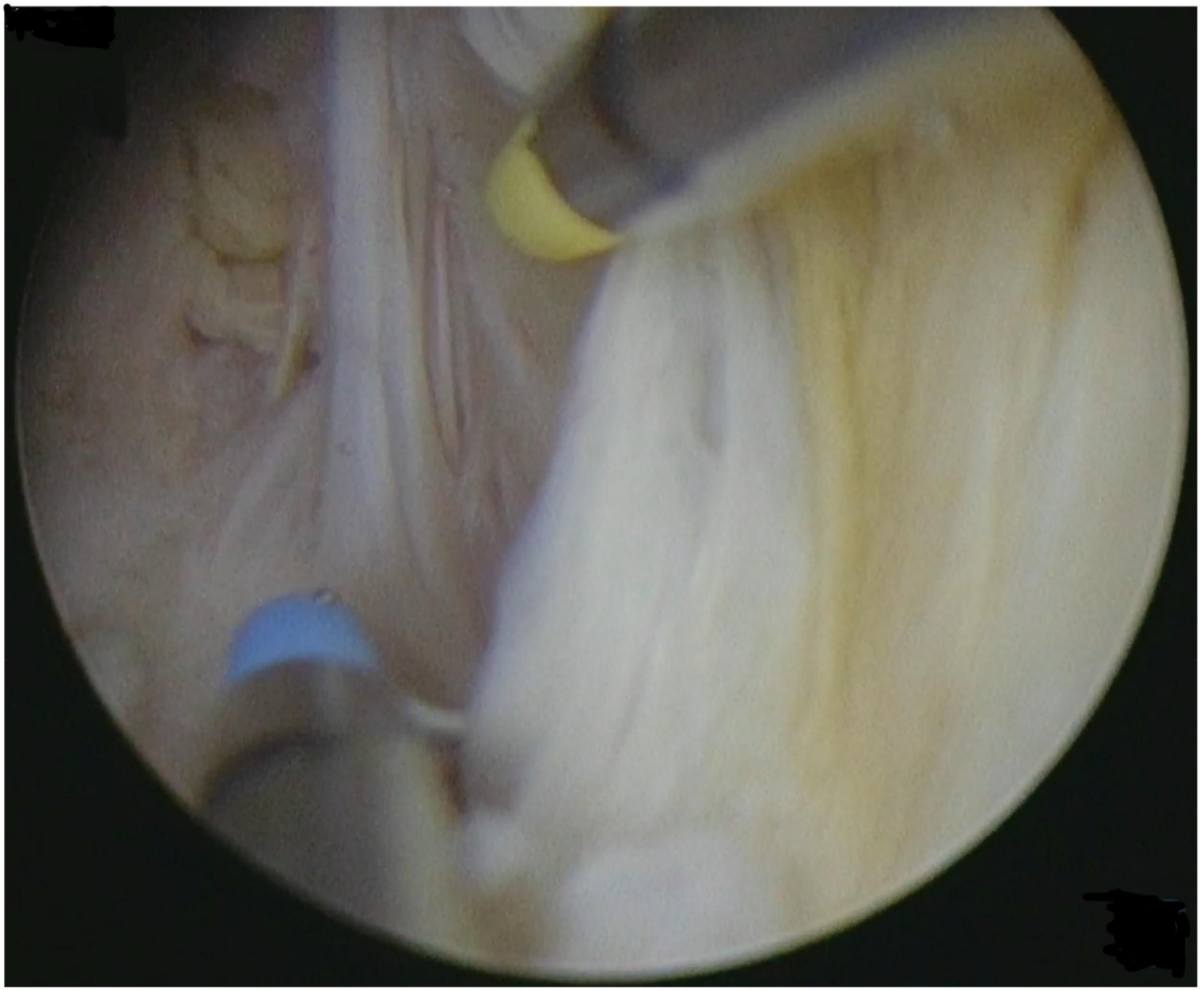
Myoma was sliced within the pseudocapsule.

**Figure 5:**
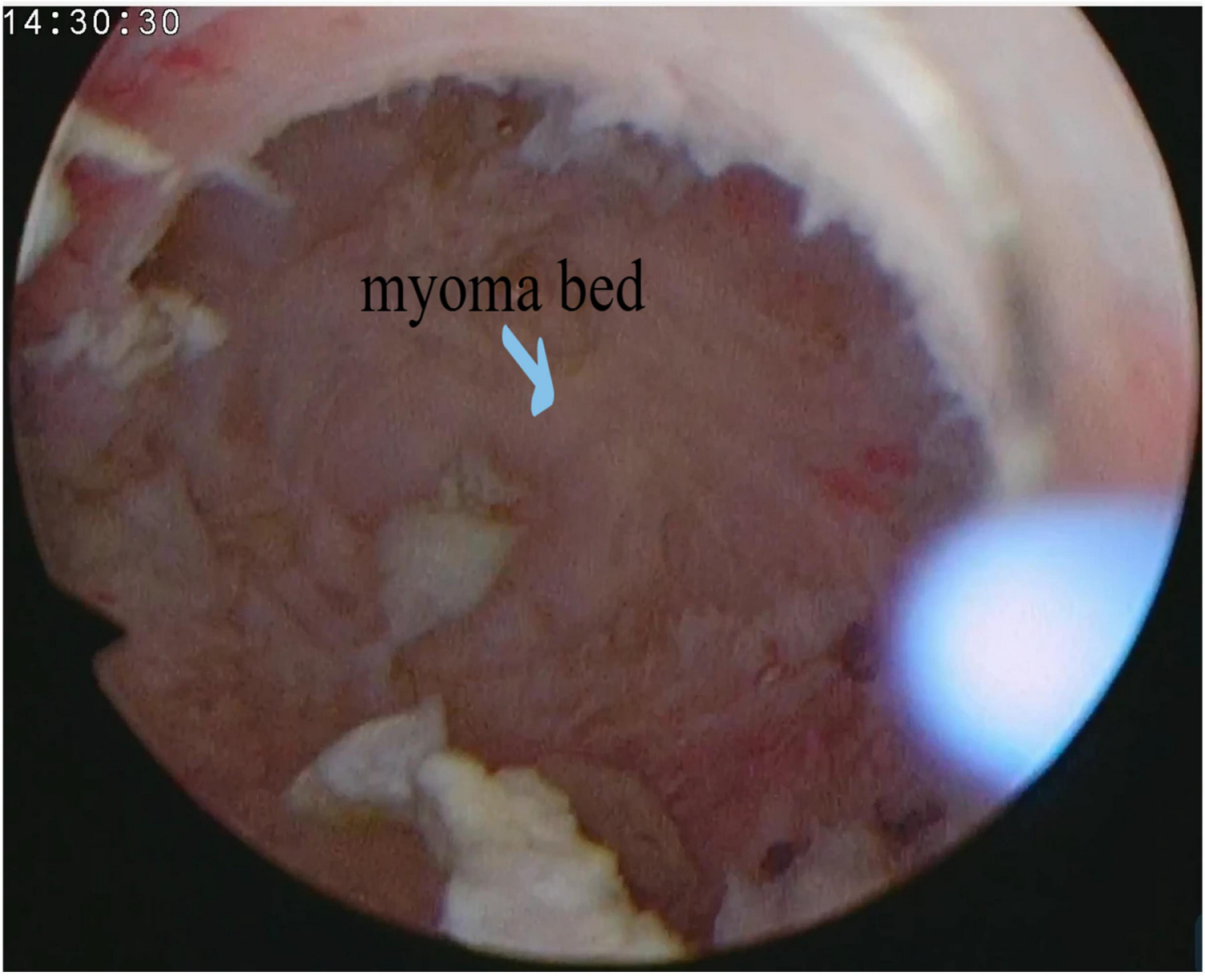
Myoma was all removed.

Type 3 myomas were initially diagnosed through preoperative 3D transvaginal ultrasound, showing myomas closely attached to the endometrium. This was confirmed by hysteroscopy, using minimal pressure to distend the uterine cavity without deformation (Figure 1). A small incision was made over the fibroid to expose its pseudocapsule (Figure 2). After opened the capsule, oxytocin was administered intravenously, meanwhile, the uterine distension pressure was lowered to 100 mmHg. The decreased intrauterine pressure and enhanced uterine contractions were all contribute to the type 3 myomas protruded into the uterine cavity. Consequently, the intramural component of the type 3 myomas gradually diminished, while the intracavitary component continuously increased. sType 3 myoma was converted into type 2 (Figure 3). Each slice was carefully made within the pseudocapsule (Figure 4). The myoma was completely resected (Figure 5).

To minimize hysteroscope insertion time and reduce the risk of trauma, fragments of the resected myoma were pushed into the fundal cavity and removed only after the cavity was filled with these fragments. Operative time was defined as the duration from hysteroscope insertion into the uterine cavity until the completion of the surgery.

During the operation, uterine distension pressure was continuously adjusted as the procedure progressed. At the start, the pressure was set to 80–100 mmHg to confirm the presence of the type 3 myomas and to allow the fibroid to sink into the uterine cavity after opening its pseudocapsule. Once most of the fibroid had been removed, the pressure was increased to 120 mmHg to ensure complete removal. Finally, the pressure was lowered to check for any bleeding in the myoma bed.

The depth of bipolar resection was also adjusted throughout the surgery. When opening the endometrium over the fibroid, thin cuts were made just enough to expose the boundary between the fibroid and its pseudocapsule. After the fibroid protruded into the uterine cavity, larger cuts were made along the inside of the pseudocapsule to quickly reduce the resection time. After most of the fibroids were removed, thin cuts were made to avoid uterine perforation.

If bleeding occurred, coagulation was applied immediately, as exposed blood vessels can increase fluid absorption. Intravenous furosemide was administered every 20 min to help prevent fluid overload. The operation was stopped immediately if signs of reduced oxygen saturation, decreased end-tidal carbon dioxide, or facial swelling appeared. The patients' vital signs and blood electrolytes were closely monitored, and fluid intake was carefully restricted for 24 h afterward.

### Measurements and follow-up

The primary outcome was the complete myoma removal rate and the presence of intrauterine adhesions, assessed using ultrasonography at 1–3 months postoperatively. Secondary outcomes included the PBAC score, duration of the menstrual period, and Hb levels, which were evaluated at 3, 6, and 12 months postoperatively.

### Statistical analyses

Continuous variables were assessed for normality using the Kolmogorov–Smirnov test. Data are expressed as mean ± standard deviation, number (%). Differences among the four independent groups were compared by Kruskal–Wallis H test, and pairwise comparisons were performed with Dunn's test with Bonferroni correction. A two-tailed *P* < 0.05 was considered statistically significant. All data were analyzed using SPSS version 25 (IBM Corp., Armonk, NY, USA).

## Results

A total of 26 women with menorrhagia or prolonged menstruation who underwent hysteroscopic myomectomy for type 3 myomas were included in this study. The mean age was 37.96 ± 6.06 years (Table 1).

**Table 1:** Description of the study population (*n* = 26).

Number of people	26
Age (years)	37.96 ± 6.06
Operative time (min)	49.19 ± 11.52
Average myoma size (cm)	2.99 ± 0.61
TURP-like syndrome, n (%)	3.84 (1)
Proportion of myoma >3 cm, n (%)	42.31 (11)
Total resection, n (%)	96.15 (25)

Data are presented as mean ± standard deviation and percentage (number). TURP, transurethral resection of the prostate syndrome.s.

The average myoma size was 2.99 ± 0.61 cm (Table 1), 42.31% (11/26) of the patients had uterine myomas larger than 3 cm, with only two patients who had myomas larger than 4 cm. The largest myoma measured 4.19 cm and was successfully resected in a single procedure. Incomplete resection occurred in one patient during the initial procedure. However, she declined a second surgery due to a noticeable reduction in menstrual bleeding postoperatively, and histopathological analysis confirmed the myoma was benign.

The mean operative time (from cervical dilatation to completion of resection) was 49.19 ± 11.52 min (Table 1). One patient developed transurethral resection of the prostate (TURP)-like syndrome. None of the patients required office hysteroscopy for follow-up, as postoperative sonography revealed no intrauterine adhesions.

The postoperative PBAC scores and duration of the menstrual period also decreased significantly in patients who experienced excessive preoperative menstrual flow or long duration of menstrual period (*p* < 0.05) (Table 2). Mean Hb levels increased significantly at 6, and 12 months postoperatively compared to baseline (*p* < 0.05) (Table 2). Three months postoperatively, the hemoglobin (Hb) level was slightly elevated compared with the preoperative baseline, though the difference failed to reach statistical significance. Additionally, three women who wished to conceive became pregnant within one year after surgery.

**Table 2:** PBAC score, duration of the period, and hemoglobin levels at baseline and at 3, 6, and 12 months after treatment.

Variable	Baseline	3 Months postoperatively (*n* = 26)	6 Months postoperatively (*n* = 26)	12 Months postoperatively (*n* = 26)	*Adj p* ^a^	*Adj p* ^b^	*Adj p* ^c^
PBAC score	110.46 ± 10.09	83.73 ± 7.10	77.77 ± 7.10	73.61 ± 7.27	*p* < 0.05	*p* < 0.05	*p* < 0.05
Duration of the menstrual period(days)	10.03 ± 1.84	7.65 ± 1.55	7.03 ± 1.22	6.53 ± 1.02	*p* < 0.05	*p* < 0.05	*p* < 0.05
Hb level (g/L)	92.23 ± 14.96	102.31 ± 12.00	109.19 ± 10.78	116.42 ± 9.81	*p* >0.05	*p* < 0.05	*p* < 0.05

Data are presented as mean ± standard deviation.

a 3 Months postoperatively compared with the baseline value.

b 6 Months postoperatively compared with the baseline value.

c 12 Months postoperatively compared with the baseline value.

## Discussion

The 2018 revision of the FIGO fibroid classification system brought greater clarity to the impact of uterine myomas on subfertility, particularly highlighting the role of type 3 myomas in compromising assisted reproductive outcomes (8, 11, 12). Surgical removal of type 3 or 4 myomas has been shown to downregulate endometrial expression of tumor necrosis factor-alpha and nuclear factor kappa B, both of which are associated with impaired endometrial receptivity (13). Type 3 myomas, although intramural, are closely abutting the endometrium and can alter endometrial receptivity through various mechanisms. These include aberrant expression and localization of matrix metalloproteinases and tissue inhibitors of metalloproteinases, as well as dysregulation of key inflammatory pathway molecules (14).

The guidelines of the International Society for Gynecologic Endoscopy recommend evaluating myomas based on size, topography, extension, penetration, and wall classification to help predict surgical complexity, the likelihood of incomplete myoma removal, prolonged operative time, fluid overload, and other major complications in patients with type 0–2 submucosal fibroids (15). For type 3 myomas, no standardized preoperative assessment tools are currently available. In our study, the single case of incomplete resection involved the largest myoma, which was located at the uterine fundus. Whether type 3 myomas can be evaluated using the same predictive models applied to type 0–2 submucosal myomas remains uncertain and warrants further investigation.

In our study, only one patient in our study failed to achieve complete resection of the uterine myoma in a single operation, resulting in an overall single-operation resection rate of 96.15% (25/26). In 2016, Capmas et al. analyzed 13 women with type 3 myomas who underwent hysteroscopic myomectomy. They reported a 31% (4/13) incomplete resections and a 37.5% rate of postoperative adhesions and, with a mean operative time of 50.38 min [95%CI, 43.58–57.19] (7). Among the study participants, 31% had type 3 uterine myomas measuring >4 cm in diameter, while the mean diameter of the myoma was 3.08 cm [95%CI, 2.38–3.80]. The possible reasons for the difference of complete resection of type 3 uterine myomas in a single operation are as follows: during the entire operation, we fully adjusted the intrauterine distension pressure to reduce the absorption of distension medium, which provided more time for the operation. Meanwhile, we aimed to resect as much of the myoma tissue as possible from within the pseudocapsule. This approach offered two key advantages: first, the pseudocapsule contains fewer exposed blood vessels, which helped minimize the amount of distension fluid absorbed into the systemic circulation; second, it allowed for the safe and efficient removal of a larger volume of myoma tissue, thereby shortening operative time. Oval forceps were used to blindly remove myoma fragments from within the uterine cavity when direct visualization was not possible. During fragment extraction, the uterine cavity was not pressurized, allowing any remaining myoma tissue to descend further into the cavity, making it accessible for subsequent resection. All these factors created conditions for the complete resection of type 3 uterine myomas in single operation. However, in Campas' study, the reasons why 4 patients failed to achieve complete resection of uterine myomas in a single operation were not reported.

Postoperative intrauterine adhesion (IUA) and pregnancy outcomes following hysteroscopic type 3 myomas myomectomy are major concerns in clinical practice. Vorona et al. later introduced an endometrial preservation technique during the resection of type 2 and 3 myomas, which involves making a minimal incision in the endometrium to expose the pseudocapsule (16). This study included only 2 cases, one case was conceived and had a successful delivery, normal endometrial development was reported in another case. In another study, a combination of cold and electrosurgical resection techniques was used to remove type 3 myomas. The rate of mild intrauterine adhesions at 3 months postoperatively was 15.4% (2/13), with no cases of moderate or severe adhesions (17). Notably, the pregnancy rate reached 84.6% (11/13), and the live birth rate was 91% (10/11) within 12–24 months (17). These outcomes suggest that precise resection within the pseudocapsule, along with minimal thermal injury, can lower the risk of adhesion formation and improve fertility outcomes. However, in our cohort, most patients did not have fertility intentions and they chose to perform 3D ultrasound rather than take office hysteroscopy at the follow-up period. Although the accuracy of 3D ultrasound in diagnosing intrauterine diseases has been continuously improving, hysteroscopy remains the gold standard for the diagnosis of IUAS. It is possible the IUAs rate was underestimated due to mild or filmy IUAs could not been detected through 3D ultrasound. Furthermore, most of the enrolled patients had either completed their childbearing plans or had no fertility requirements in the short follow-up period. Only 3 patients were reported to had achieved pregnancy. This study is insufficient to prove the positive impact of type 3 uterine myoma resection on pregnancy, which should be further evaluated in a larger population of women desiring future pregnancy.

A case in our study suffered TURP-like syndrome, which appears to occur more frequently during hysteroscopic surgery for type 3 myomas. Careful fluid management is crucial. A fluid deficit of up to 1,000 mL during bipolar myomectomy using saline is generally considered low risk for TURP syndrome (15). However, deficits exceeding 1,000 mL require close monitoring and may necessitate early termination of the procedure. Intravenous injection of oxytocin facilitated the descent of myometrial fibroids into the uterine cavity, allowing for rapid resection. However, in the case of the patient who developed TURP-like syndrome, oxytocin was administered via cervical injection, which likely caused an abnormal increase in intrauterine pressure. This, in turn, may have led to the entry of distension fluid into the circulatory system. During the procedure, the patient's blood oxygen saturation dropped, raising suspicion of pulmonary edema and fluid overload. Therefore, the surgery was immediately halted, and the patient was treated with high-flow oxygen, diuretics, and electrolyte correction. Her condition stabilized, and she recovered fully by the following day.

Menstrual flow decreased significantly in patients following surgery. Uterine myomas are monoclonal tumors originating from the myometrium and can have widespread effects on the endometrium. They produce excess extracellular matrix components, contributing to their stiffness and growth. Myomas disrupt normal uterine functions, such as menstrual bleeding, endometrial receptivity, and implantation, because of changes in mechanotransduction, resulting in decreased uterine wall contractility and increased myometrium rigidity (18). These pathophysiological changes help explain the improvement in menstrual bleeding observed after resection of type 3 myomas.

This study has some limitations. This was a retrospective, single-center study with a small number of cases. Therefore, large-scale, prospective, randomized studies with longer follow-up periods are required to validate our results. Most of the patients in the study were multiparous with no fertility requirements. Whether the endometrial receptivity can be improved after the removal of type 3 myomas requires further research.

In conclusion, this is a retrospective study conducted at a single center, involving 26 patients with single type 3 myomas who underwent hysteroscopy resection of type 3 myomas within pseudocapsule with endometrial preservation. We preliminarily conclude that this technique is feasible and safe in this highly selected patient population.

## Data Availability

The raw data supporting the conclusions of this article will be made available by the authors, without undue reservation.
